# Supplementary material for: Efficient, Recyclable, and Heterogeneous Base Nanocatalyst for Thiazoles with a Chitosan-Capped Calcium Oxide Nanocomposite
Source: Polymers (Basel). 2022 Aug 17;14(16):3347. doi: 10.3390/polym14163347 (PMC9416520; doi:10.3390/polym14163347)
Supplement: Supplementary file 1 [file polymers-14-03347-s001.zip › polymers-1804124-supplementary.pdf]

Supplementary data

Efficient, Recyclable, Heterogeneous Base Nanocatalyst for Thiazoles with a Chitosan-Capped Calcium Oxide Nanocomposite

Khaled D. Khalil[1,2]*, Hoda A. Ahmed[1], Ali H. Bashal[2] , Stefan Bräse [3,4],*, AbdElAziz A. Nayl[5], Sobhi M. Gomha[6,1]*

[1] Department of Chemistry, Faculty of Science, Cairo University, Giza, 12613, Egypt.
[2] Department of Chemistry, Faculty of Science, Taibah University, Al-Madinah Almunawarah, Yanbu, 46423, Saudi Arabia.
[3] Institute of Organic Chemistry (IOC), Karlsruhe Institute of Technology (KIT), Fritz-Haber-Weg 6, 76133 Karlsruhe, Germany.
[4] Institute of Biological and Chemical Systems (IBCS-FMS), Karlsruhe Institute of Technology (KIT), Eggenstein-Leopoldshafen, Germany.
[5] Department of Chemistry, College of Science, Jouf University, P.O. Box 2014, Sakaka, Aljouf, Kingdom of Saudi Arabia,
[6] Department of Chemistry, Faculty of Science, Islamic University of Madinah, Madinah 42351, Saudi Arabia.

* Correspondence: khd.khalil@yahoo.com (KDK), stefan.braese@kit.edu (S.B.) smgomha@iu.edu.sa (SMG)

2.1. Apparatus, Instrumentations and Materials

Melting points of the synthesized compounds were obtained using an electrothermal Gallenkamp equipment (*GallenKamp, Lister, United Kingdom*) and are uncorrected. Fourier transform infrared spectra (FTIR) with a Nicolet Magna 6700 FT spectrometer (*Thermo Fisher Scientific, Waltham, M.A.United States*) were conducted in a wavenumber region (500–4,000 cm^{-1}). The mass spectra were measured on a GCMSQ1000-EX Shimadzu and GCMS 5988-A HP spectrometers (*Shimadzu, Kyoto, Japan*) with a 70-eV ionizing voltage. The ^{1}H- and ^{13}C-NMR spectrums were recorded on a Varian Mercury VXR-300 spectrometer (300 MHz for ^{1}H-NMR and 75 MHz for ^{13}C-NMR) and the chemical shifts were related to that of the solvent DMSO-d_6 (*Varian, Inc., Karlsruhe, Germany*). For SEM and EDX (*HRSEM, JSM 6510A, Jeol Ltd., Tokyo, Japan*) measurements, the thin films were cut into small pieces and put on the SEM stubs with carbon tape. Then the samples were coated with 4 nm thickness of platinum layer, after that transferred into SEM Teneo/Quattro for imaging. Images were taken under high vacuum with different magnifications. X-ray diffraction (XRD) patterns were studied using a Philips diffractometer (*Model: X'Pert-Pro MPD; Philips, now PANaytical, Malvern, Worcestershire, United Kingdom*) with Cu Kα radiation (wavelength 1.5418 Å) at 40 kV and 40 mA. The

patterns were collected between 2θ of 10° and 40°, and the scan speed was 1.5 degree/min. Sonication was performed in Shanghai Branson-CQX ultrasonic cleaner *(Ecoclean Machinery (Shanghai) Co., Ltd., China)* at frequency of 40 kHz and ultrasonic power was kept at 250 W. Sodium hydroxide was purchased from Sigma-Aldrich Company. Triple distilled water was used in all solution preparations. Chitosan was provided by Sigma Aldrich (Shanghai, China) (powder, medium molecular weight, shrimp shells source, batch no. C3646, density = 0.15–0.3 g/cm^3). Calcium oxide (nanopowder < 160 nm particle size (BET) 98%, product no. 634182) was purchased from Sigma-Aldrich. Sodium hydroxide (ACS reagent, ≥97.0%, pellets; product no. 221465), potassium hydroxide (reagent grade, 90%, flakes, product no. 484016), methanol (ACS reagent, ≥99.8%; product no. 179337), and acetic acid (glacial, ACS reagent, ≥99.7%; product no. 695092), were purchased by middle east supplier of Merck Company (Rahway, NJ, USA) and were used as such without further purification.

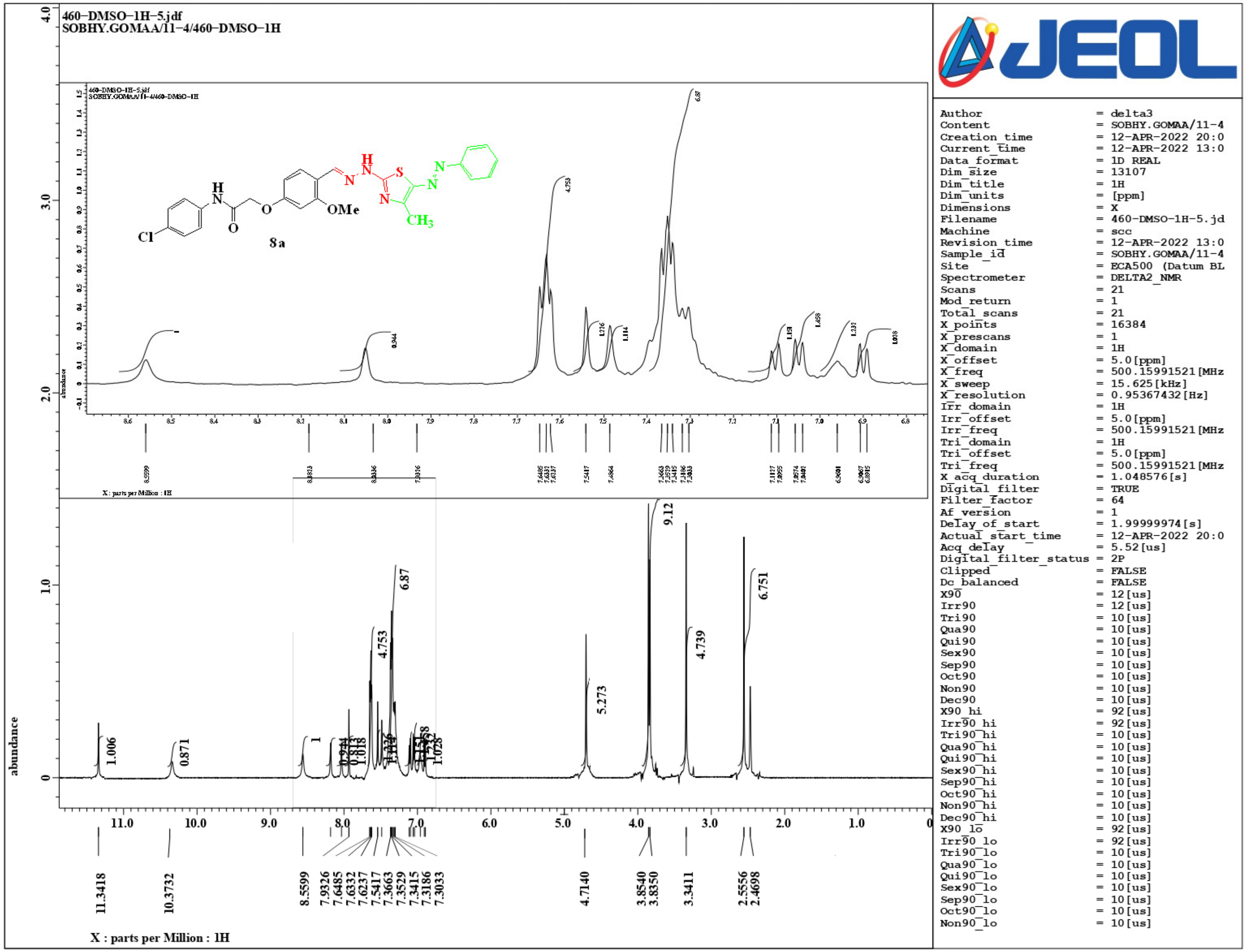

Figure S1. ¹H-NMR spectrum of compound **8a**

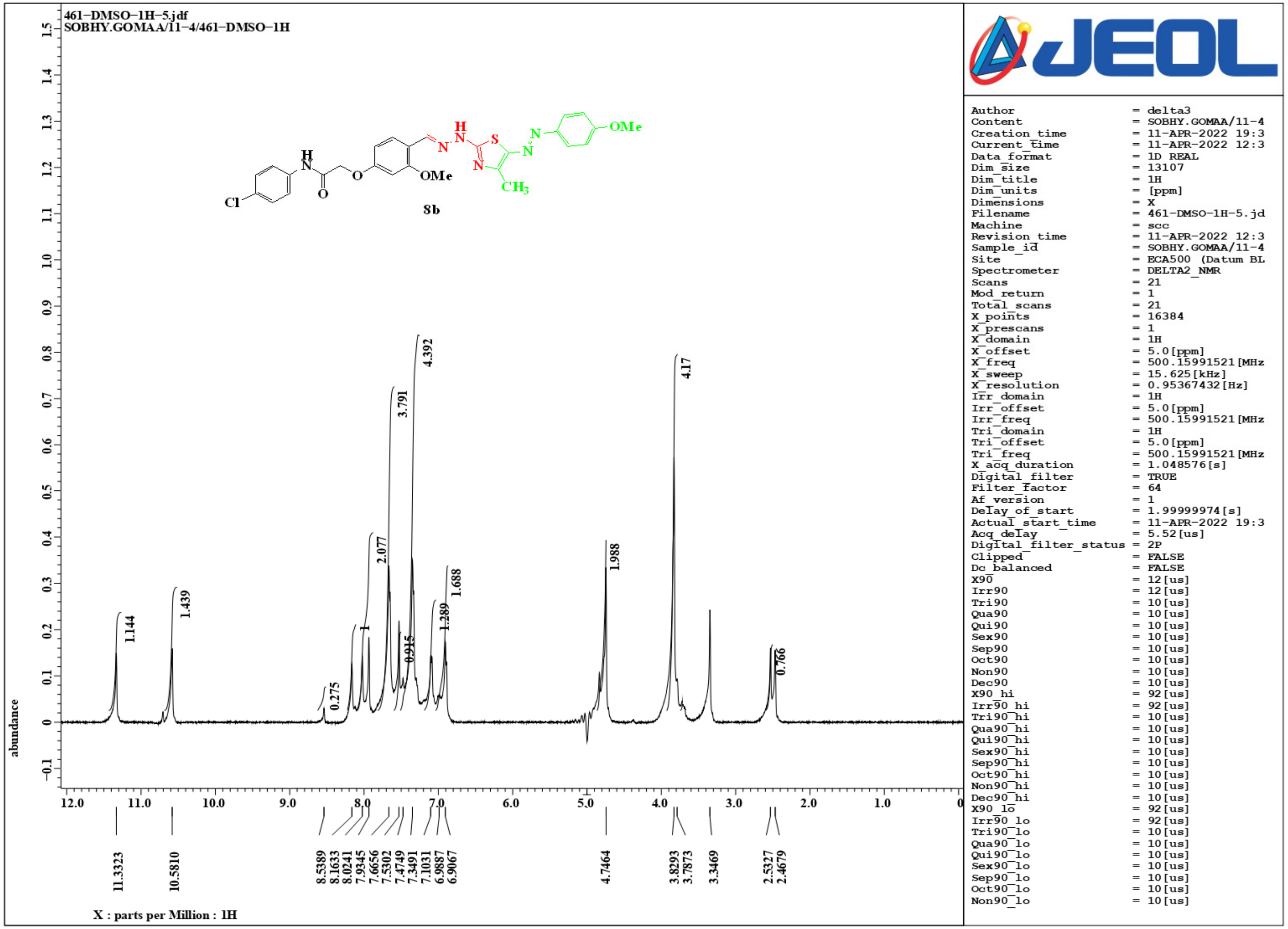

Figure S2. ^{1}H-NMR spectrum of compound **8b**

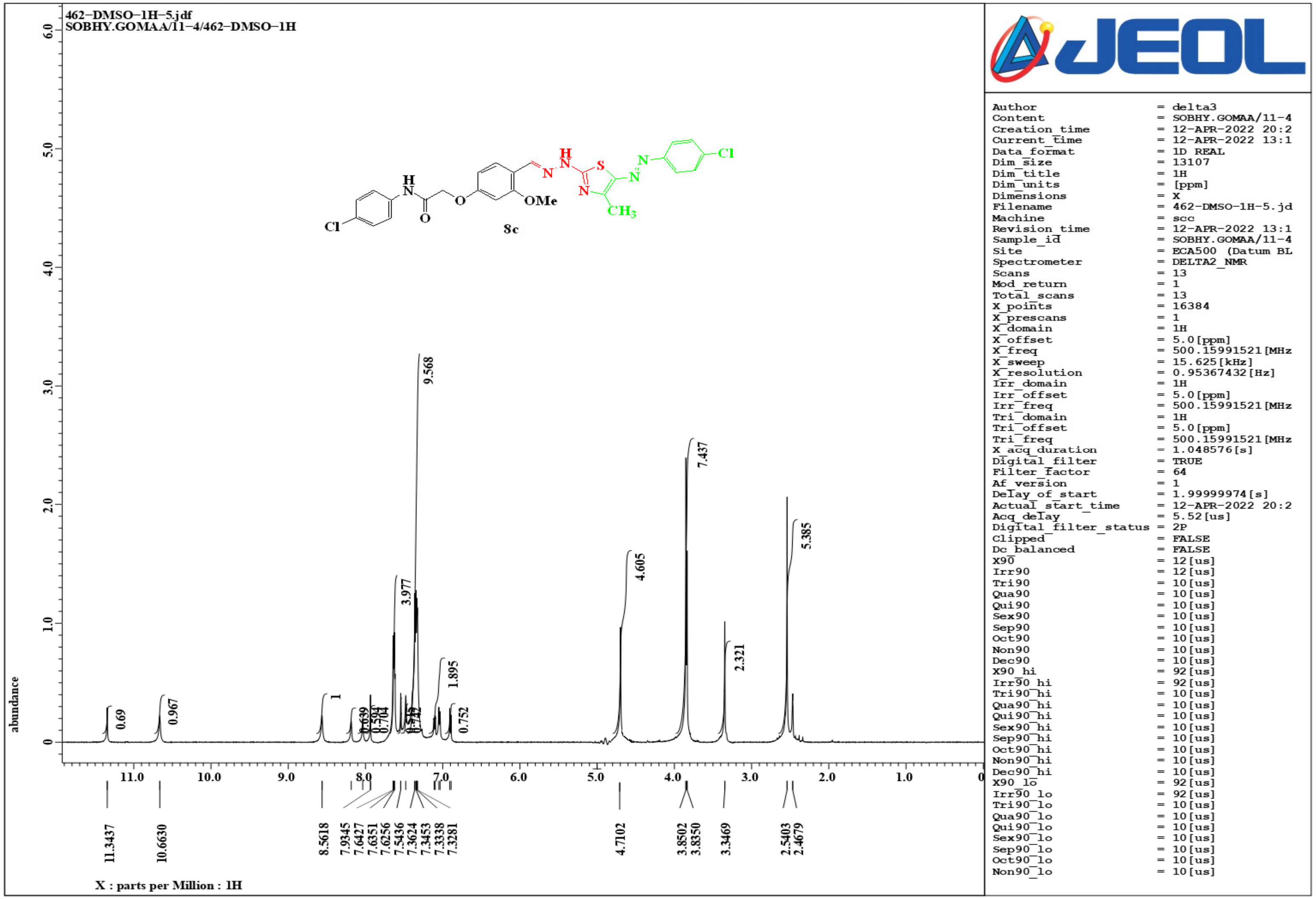

Figure S3. ^{1}H-NMR spectrum of compound **8c**

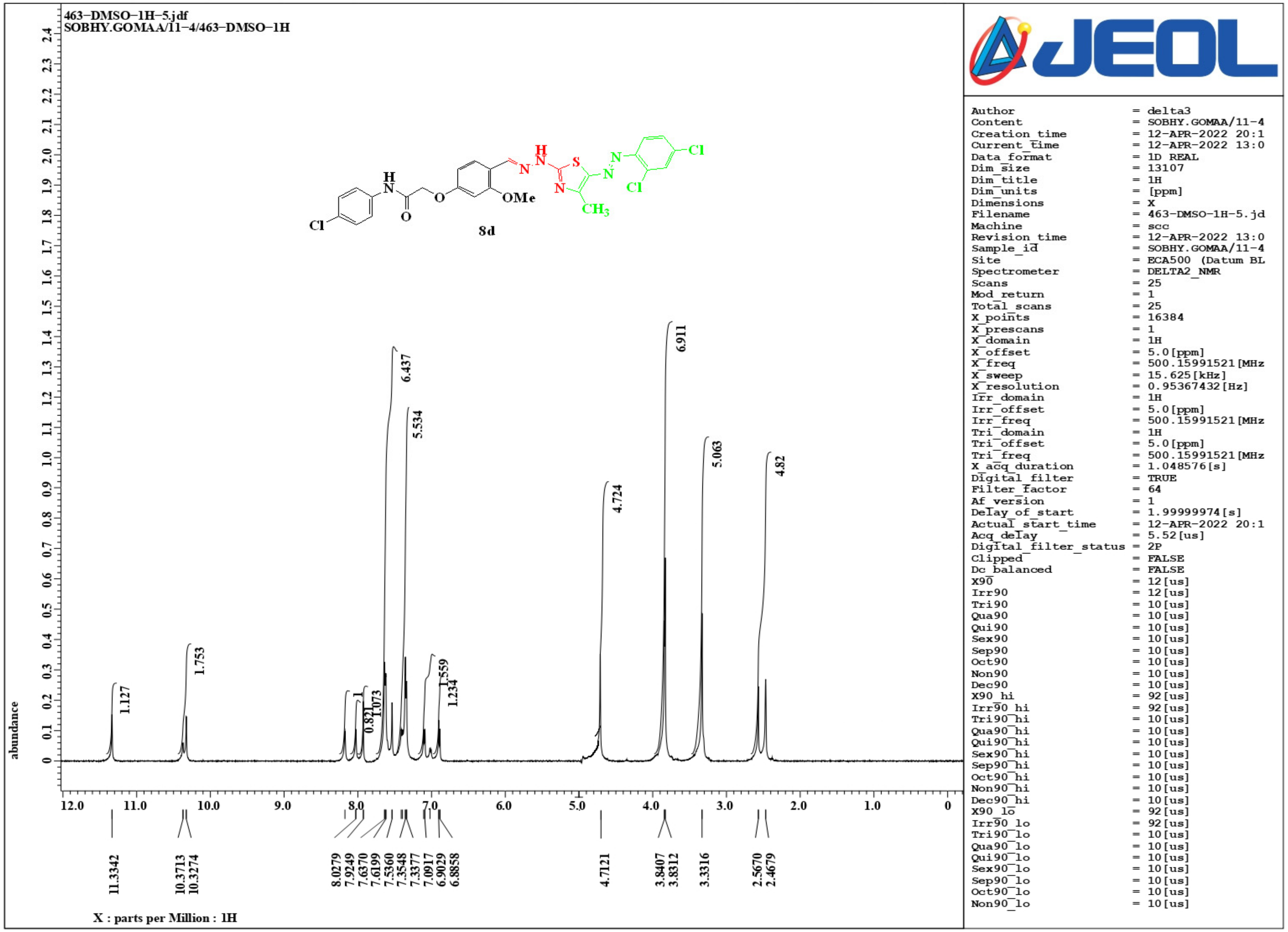

Figure S4. ^{1}H-NMR spectrum of compound **8d**

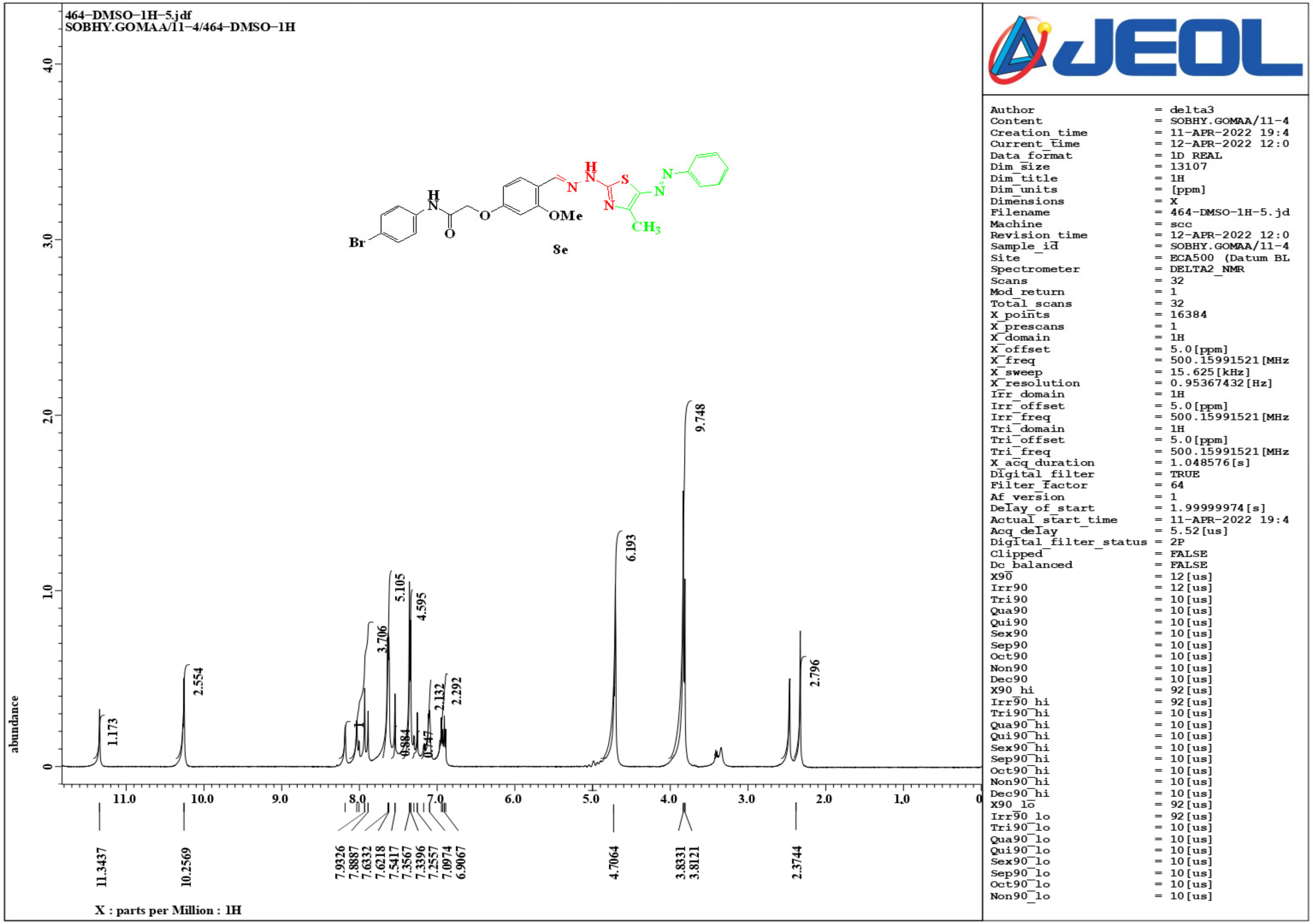

Figure S5. ^{1}H-NMR spectrum of compound **8e**

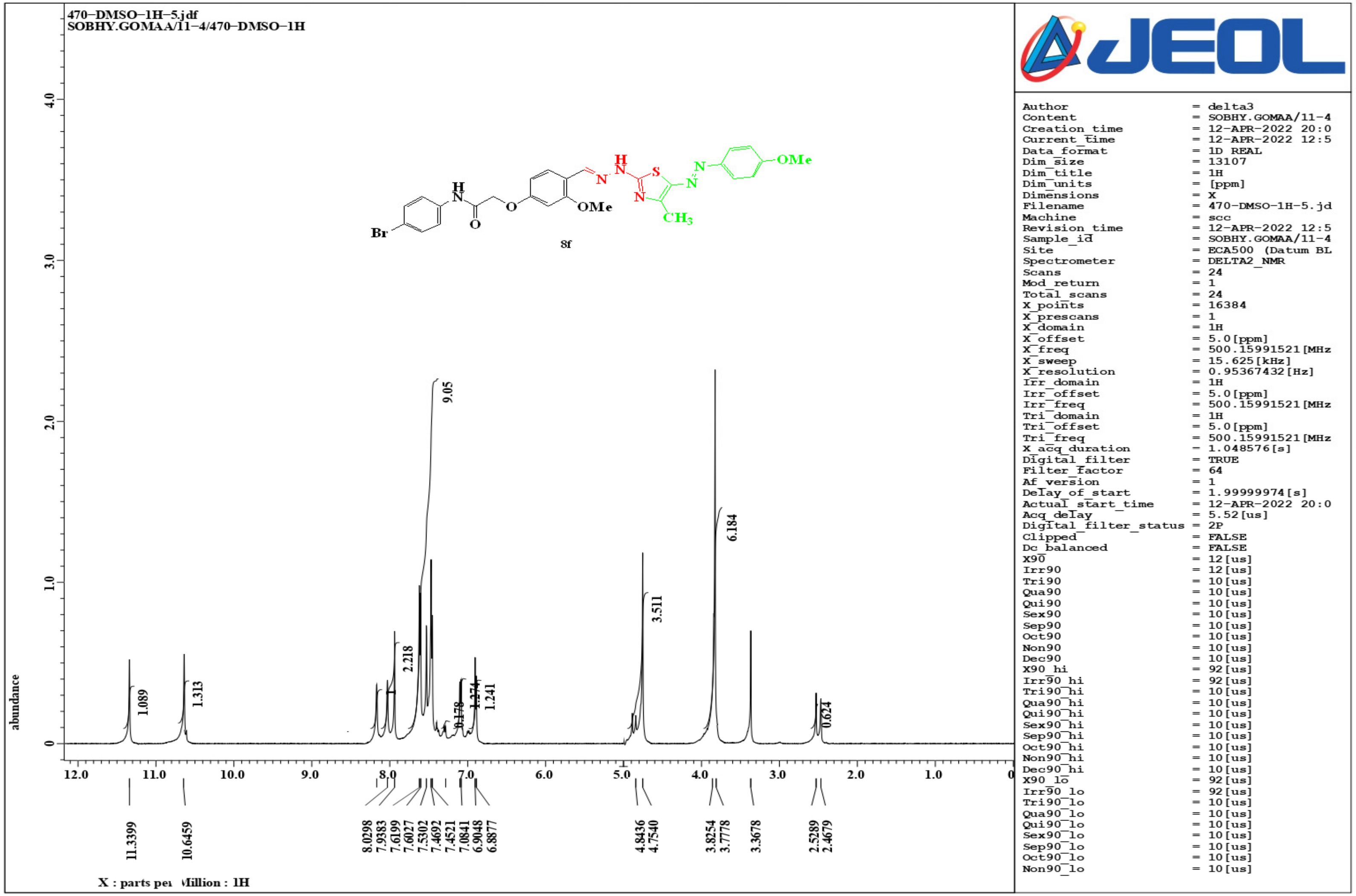

Figure S6. ^{1}H-NMR spectrum of compound **8f**

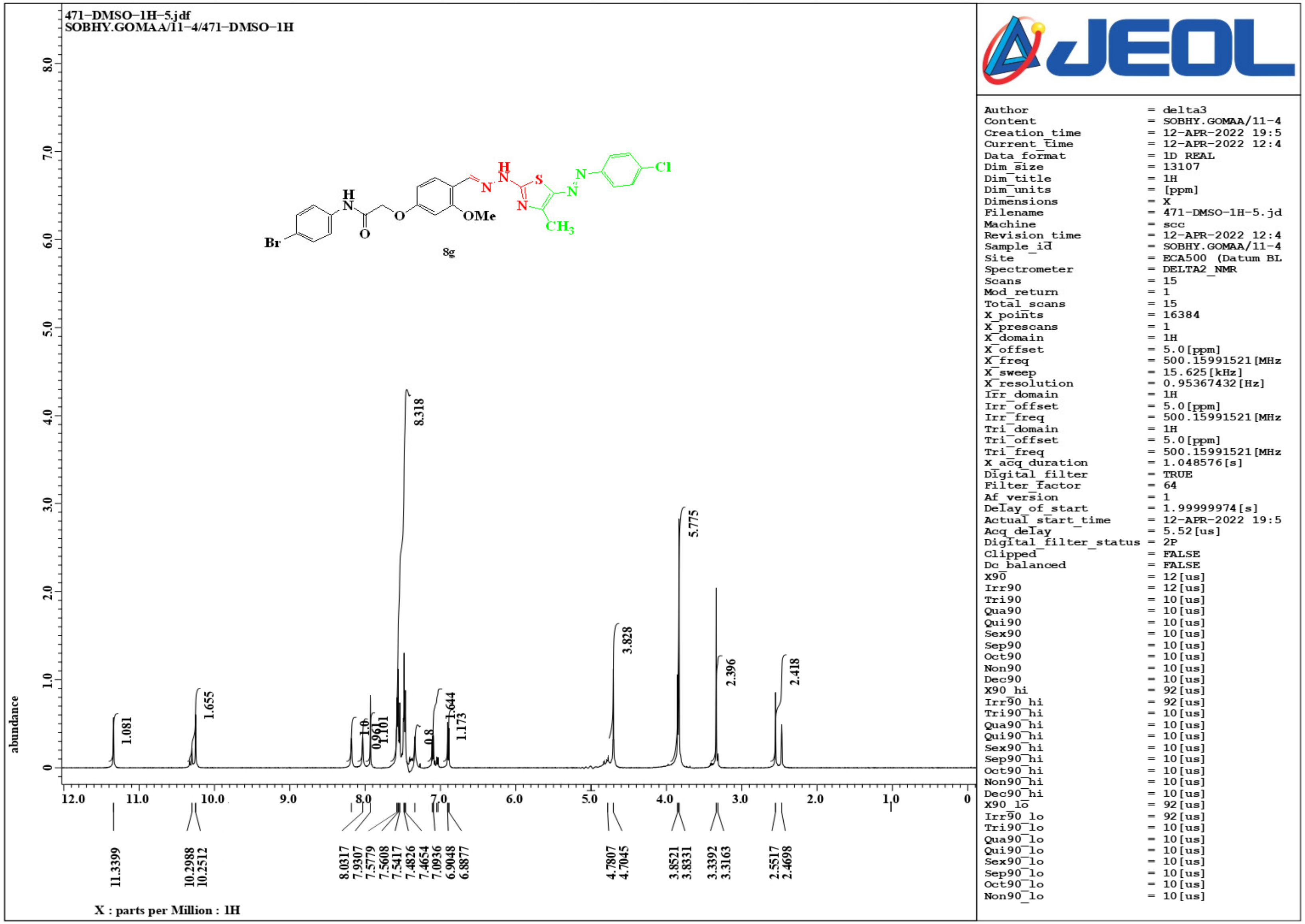

Figure S7. ^{1}H-NMR spectrum of compound **8g**
